# Site Saturation Mutagenesis Demonstrates a Central Role for Cysteine 298 as Proton Donor to the Catalytic Site in CaHydA [FeFe]-Hydrogenase

**DOI:** 10.1371/journal.pone.0048400

**Published:** 2012-10-25

**Authors:** Simone Morra, Alberto Giraudo, Giovanna Di Nardo, Paul W. King, Gianfranco Gilardi, Francesca Valetti

**Affiliations:** 1 Department of Life Sciences and Systems Biology, University of Torino, Torino, Italy; 2 Biosciences Center, National Renewable Energy Laboratory, Golden, Colorado, United States of America; Queen's University Belfast, United Kingdom

## Abstract

[FeFe]-hydrogenases reversibly catalyse molecular hydrogen evolution by reduction of two protons. Proton supply to the catalytic site (H-cluster) is essential for enzymatic activity. Cysteine 298 is a highly conserved residue in all [FeFe]-hydrogenases; moreover C298 is structurally very close to the H-cluster and it is important for hydrogenase activity. Here, the function of C298 in catalysis was investigated in detail by means of site saturation mutagenesis, simultaneously studying the effect of C298 replacement with all other 19 amino acids and selecting for mutants with high retained activity. We demonstrated that efficient enzymatic turnover was maintained only when C298 was replaced by aspartic acid, despite the structural diversity between the two residues. Purified CaHydA C298D does not show any significant structural difference in terms of secondary structure and iron incorporation, demonstrating that the mutation does not affect the overall protein fold. C298D retains the hydrogen evolution activity with a decrease of *k*_cat_ only by 2-fold at pH 8.0 and it caused a shift of the optimum pH from 8.0 to 7.0. Moreover, the oxygen inactivation rate was not affected demonstrating that the mutation does not influence O_2_ diffusion to the active site or its reactivity with the H-cluster. Our results clearly demonstrate that, in order to maintain the catalytic efficiency and the high turnover number typical of [FeFe] hydrogenases, the highly conserved C298 can be replaced only by another ionisable residue with similar steric hindrance, giving evidence of its involvement in the catalytic function of [FeFe]-hydrogenases in agreement with an essential role in proton transfer to the active site.

## Introduction

[FeFe]-hydrogenases are a class of redox enzymes able to catalyse both hydrogen evolution and uptake [1], according to the simple reaction 2H^+^ + 2e^−^ ↔ H_2_, with a bias towards hydrogen evolution. These enzymes are widely distributed in several microorganisms, both prokaryotic and eukaryotic and are involved in energy metabolism [2]. Moreover, their peculiar catalytic activity can be exploited to produce molecular hydrogen in industrial processes, either using microorganisms [3], [4] or bio-inspired artificial devices [5]–[7].

**Figure 1:**
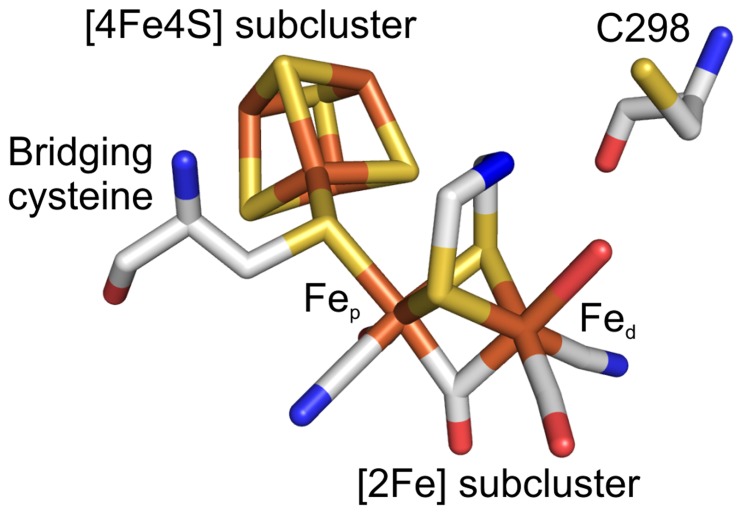
Structure of the H-cluster. The organometallic cluster is covalently linked to the protein by the bridging cysteine. Cysteine 298 (C298) is located very close to the organic ligand and to the catalytically active site Fe_d_. Fe atoms are orange, S atoms yellow, C atoms grey, N atoms blue and O atoms red.

The [FeFe]-hydrogenases have a modular structure consisting of a conserved catalytic domain and in some cases an accessory domain functioning in electron transfer [8], [9]. The catalytic domain hosts the active site organometallic cluster (H-cluster) (Figure 1) that is composed of two subclusters: a cubane [4Fe–4S] bridged to a unique [2Fe] subcluster via a conserved cysteine [10], [11]. The [2Fe] subcluster is composed of two iron atoms, a proximal Fe_p_ and a distal Fe_d_, coordinated by non-protein ligands that are three CO, two CN [12] and bridged by an organic ligand proposed as a di(thiomethyl)amine [13], although an O assignment has also been proposed instead of N as the bridging atom of the dithiolate ligand [12].

Because of its unique structure, the H-cluster requires a dedicated set of assembly enzymes for its biosynthesis involving a complex mechanism that has not yet been fully elucidated [14], [15].

On the basis of the available 3D crystal structures [10], [11] and several spectroscopic studies [16]–[18] the H-cluster structure, as well as the maturation process for biosynthesis and delivery, is considered to be the same in all [FeFe]-hydrogenases. Moreover, the active site environment is highly conserved, as demonstrated by multiple alignments of hundreds of known sequences [19].

Catalytic activity occurs on the [2Fe] subcluster, particularly on the Fe_d_ [16], [13], where reversible H_2_ activation is allowed by electron transfer with the [4Fe–4S] subcluster. During H_2_ evolution, protonation of Fe_d_ is considered a required step in the [FeFe]-hydrogenase catalytic cycle. The active site determinants that function in the accompanying proton transfer have not yet been formally characterized.

The cysteine located adjacent to the bridgehead group of the H-cluster (Figure 1), position C298 in Clostridium acetobutylicum CaHydA, is a strictly conserved residue in all functional [FeFe]-hydrogenases [2], [19]. On the basis of the CpI crystal structure the cysteine was suggested to participate in proton transfer during catalysis, transferring protons from a molecule of structural water to the active site [10], [20]. Computational simulations have supported this hypothesis [21], [22].

Only recently it was experimentally demonstrated by site directed mutagenesis that replacement of C298 in CaHydA [23], or its corresponding site in Clostridium pasteurianum CpI [24], [19] and Chlamydomonas reinhardtii CrHydA1 [19] with alanine, leucine or serine severely impaired or fully abolished catalytic activity. Moreover, FTIR and EPR studies of the cysteine-to-serine exchange in CrHydA1 showed that the H-cluster of the inactivated enzyme had equilibrated in the H_trans_ redox state [19]. Taken together, these results demonstrated that the conserved active site cysteine has an essential role in [FeFe]-hydrogenase catalysis.

Here, site saturation mutagenesis of the conserved cysteine C298 is used to investigate the specific role of this residue in Clostridium acetobutylicum CaHydA [FeFe]-hydrogenase. This technique randomly replaces this single residue with all possible 20 amino acids and the effect of each mutation is then simultaneously assayed by activity screening. Site saturation mutagenesis combines the advantages of rational design and directed evolution [25]. This approach allows all possible variants to be studied at the same time, thus immediately giving much information on the role of the targeted residue and on the effect of each replacement. Statistical analysis confirmed that the number of screened clones gives a complete coverage, allowing a detailed analysis of the mutational space at this position and providing experimental evidences on cysteine 298 role in [FeFe]-hydrogenases catalytic mechanism.

## Results

### Site saturation mutagenesis of C298

**Figure 2:**
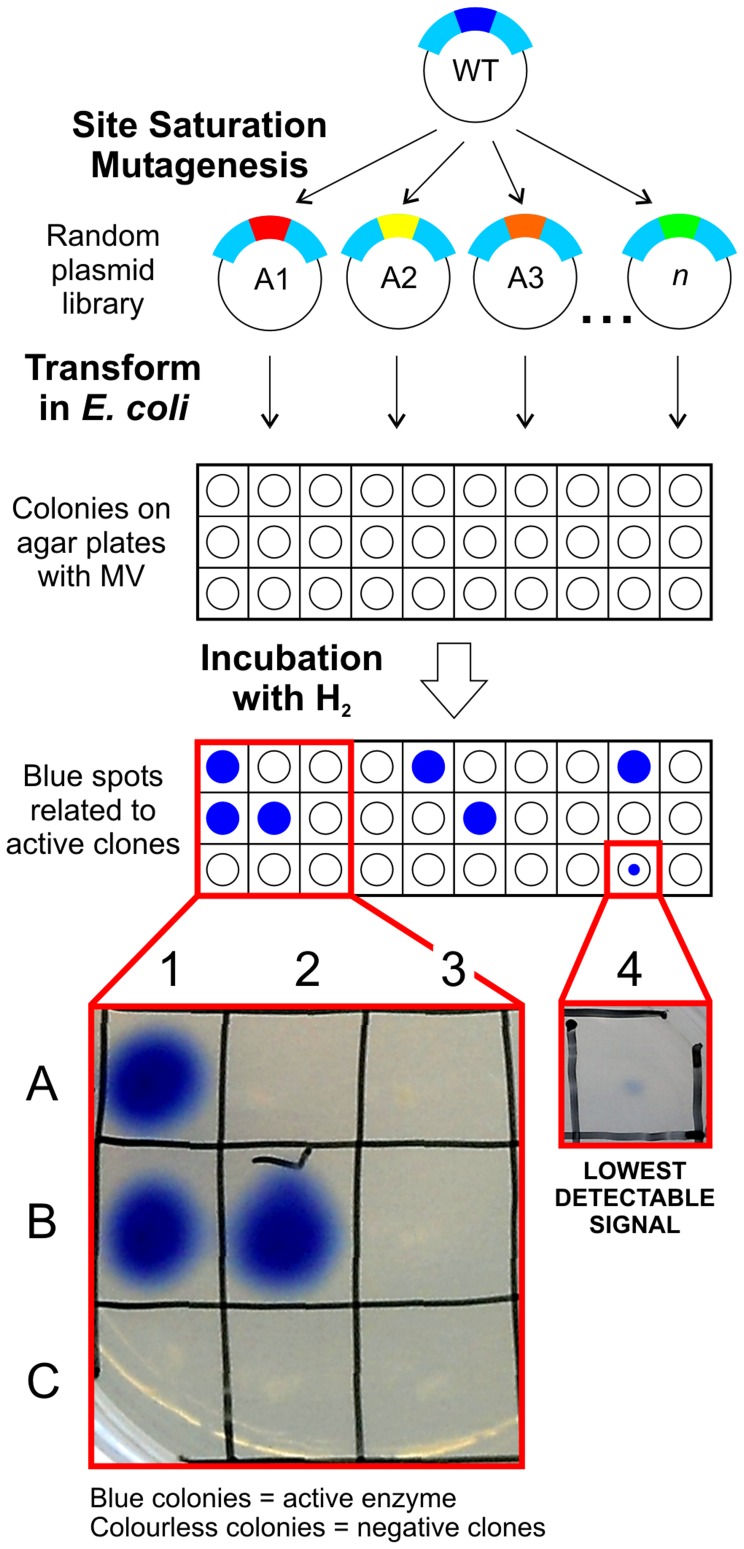
Scheme of C298 site saturation library screening. Each clone from the library is tested for hydrogenase activity *in vivo* in *E. coli* colonies. Only colonies expressing an active variant specifically developed a blue colour due to H_2_-driven reduction of methyl viologen (MV). Inset: Representative set of *E. coli* colonies used. Most clones are negative (colourless), while some (blue) show *wild type*-like activity (A1, B1). As positive control, a colony expressing CaHydA *wild type* was also plated (B2).

A library of CaHydA where the codon for C298 was replaced by the degenerate codon NNK was obtained and screened for hydrogenase activity. For this purpose, we developed a specific *in vivo* recombinant screening method in which hydrogenase activity (hydrogen uptake) of each variant was rapidly and semi-quantitatively assayed directly on *E. coli* colonies, as described in Figure 2. Colonies expressing an active hydrogenase generated a blue colour when methyl viologen (MV) is present in the medium and molecular hydrogen is added.

The sensitivity level of the screen was tested by assaying another mutant available in the laboratory with known activity. The purified mutant enzyme has 14% of WT activity for hydrogen uptake and 18% of WT activity for hydrogen evolution and shows the lowest detectable signal in our screening method (Figure 2). This gives the estimation of the lowest threshold activity detectable with the method.Library screening showed (Figure 2) that the majority of the clones did not display any detectable hydrogenase activity (negative clones), while some had a full wild type-like activity. No clones with an intermediate activity were identified.

**Figure 3:**
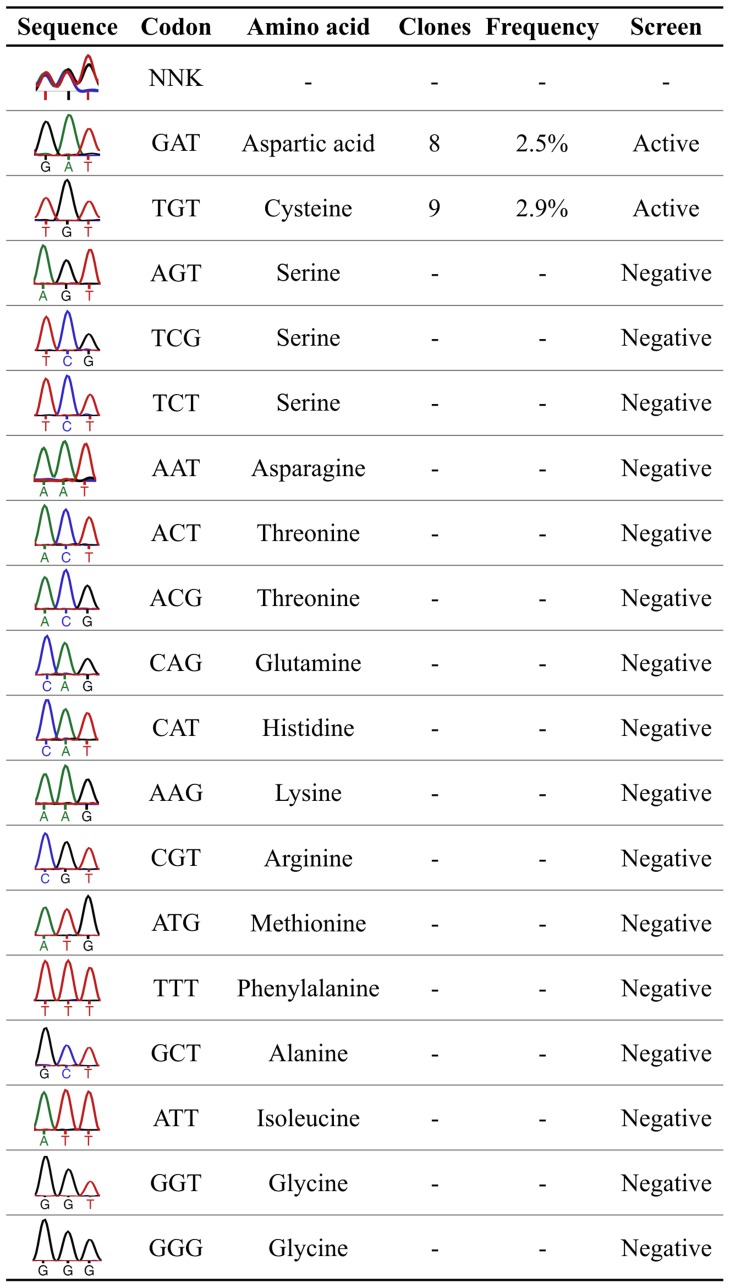
Sequencing of the randomized library, of all active clones and of a representative subset of negative clones. DNA sequencing revealed that only aspartic acid, encoded by the codon GAT, was able to functionally replace cysteine 298. Back mutations to cysteine were also observed. Frequencies (for active clones only) were calculated as the ratio between the number of clones with the specific codon and the total number of screened clones. The results of the sequencing of the whole randomised library and of a representative subset of negative clones are shown.

DNA sequencing (Figure 3) revealed that the C298 codon was randomized as expectedand that all highly active clones (screening signal comparable to WT) contained the codon GAT (aspartic acid) or TGT (back mutation to the wild type cysteine). To further confirm the diversity of residues contained in the library, a representative subset of negative clones was sequenced, showing a number of codons coding for several other different aminoacids (Figure 3).

NNK degeneracy ensures that all 20 amino acids are encoded [26], with a total number of possible DNA variants that equals 32; each codon has an expected frequency of 3.1% (1 out of 32). The observed frequency of variants (Figure 3) was 2.5 to 2.9%, which was in good agreement with the expected value. In fact aspartic acid can be encoded only by the GAT codon and cysteine only by TGT codon.

The statistics of the random mutagenesis was determined according to the following equation [27]:
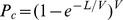
where *P_c_* is the probability that every variant is represented in the library, *L* is the number of clones used and *V* is the total number of possible DNA variants.

The library size was 360 clones, giving a *P_c_* of the library of 99.96%; 315 clones were screened, giving a *P_c_* of the screening of 99.83%. Thus, the overall probability that each variant was both represented and tested was the combinatorial product of the two above, or *P_c_* = 99.79%. This ensures a high level of confidence on the full coverage of the screening method, i.e. all 20 amino acid variants have been screened at least once.

Site saturation mutagenesis demonstrates that the enzyme maintains high catalytic activity and efficiency comparable to WT only when C298 is functionally replaced by D298 while all other variants do not show any detectable activity, implying that the activity is below the detection limit (14% of WT).

### Characterization of CaHydA WT and C298D

Proteins with C-terminal Strep-TagII were recombinantly expressed in *E. coli* and purified on affinity column. All manipulations were carried out under strict anaerobic conditions, to avoid oxygen inactivation. The final yield was 2.5 mg pure protein/L culture for both WT and C298D mutant.

To support the results of the library screening, kinetics of hydrogen evolution activity was studied by gas chromatography using methyl viologen as artificial electron donor (Table 1); C298D shows a slightly higher *K*_M_ for reduced methyl viologen, while *k*_cat_ is lowered by 47% when compared to the WT. This demonstrates that the mutant is still able to catalyse hydrogen evolution at high rate (242±9 s^−1^).

**Table 1:** Hydrogen evolution characterization.

	*K*_M_ (mM)	*k*_cat_ (s^−1^)
**WT**	6.4±0.9	456±20
**C298D**	9.7±0.9	242±9

Kinetic parameters calculated with reduced methyl viologen as artificial electron donor at 37°C in 50 mM Tris·HCl pH 8.0.

The oxygen inactivation rate is not influenced by the C-to-D mutation: the half-life of CaHydA WT exposed to air was 7.87±0.23 min, while that of C298D was 7.86±0.12 min.

**Figure 4:**
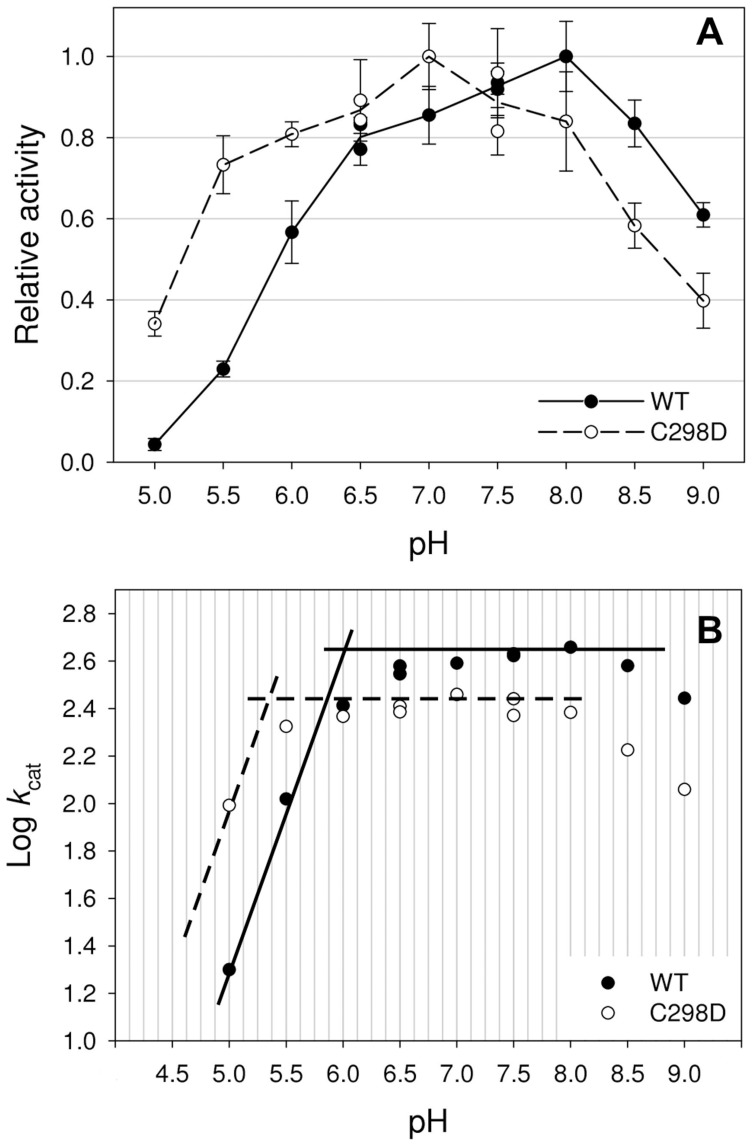
A) pH activity profile of CaHydA WT (full line) and C298D (dashed line). Hydrogen evolution activity was assayed in the pH range 5.0–9.0 using appropriate buffering agents at 37°C, with 10 mM reduced methyl viologen as artificial electron donor. Relative activity was calculated as the ratio with the maximum activity. **B) Dixon-Webb Plot for Log** ***k*** **_cat_** ***vs*** **pH activity profile of CaHydA WT (full line) and C298D (dashed line).** The datapoints at higher pH (8.5–9.0) were not fitted to the Dixon-Webb Model since effects due to low proton concentration compared to the enzyme concentration (nM) are expected to occur in this region.

Moreover, since cysteine and aspartic acid are both ionisable residues, the effect of pH on the enzymatic activity was studied by measuring hydrogen evolution over a pH range from 5.0 to 9.0 (Figure 4a). The resulting profiles show that while CaHydA WT has an optimum at pH 8.0 and its activity is almost fully lost at pH 5.0, the CaHydA C298D profile is significantly shifted by 1 pH unit towards a more acidic pH value, showing an optimum pH at 7.0. The activity of C298D at higher pH is significantly lower than that of the WT enzyme, whereas at the lower pH values, C298D retained significantly higher activity. The non-linear fitting to a sigmoidal curve performed on the data obtained for *k*_cat_ values dependence on pH between 5 and 8 highlighted a flexus point at 5.89±0.05 for WT (r^2^ = 0.99) and at 5.12±0.08 for C298D (r^2^ = 0.90). In order to provide more insight into the ionization changes associated with the C298D mutation, data were also analysed (Figure 4b) according to a Dixon –Webb Plot [28]. Log *k*_cat_ profiles show pK_a_ values of groups that must be correctly protonated/deprotonated for catalysis [29]. The slope of 1.0 displayed for both WT and mutant is consistent with the titration of a single ionisable group, while the points of intersection indicate the WT and C298D pK_a_, with a shift of about 0.7 towards more acidic pH upon C to D mutation and with values in accordance with the calculated flexus points.

**Figure 5:**
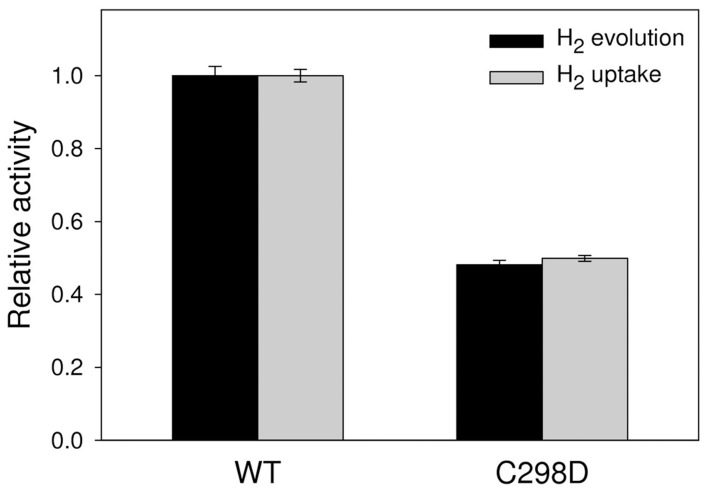
Hydrogenase activity assays. Enzymatic activity was assayed both in the direction of hydrogen evolution and hydrogen uptake at pH 8.0 with 10 mM methyl viologen as electron mediator, showing that C298D causes an equal decrease in the reaction rate for both reactions. Relative activity was calculated as the ratio with *wild type* activity (H_2_ evolution: 230±6 µmol H_2_ min^−1^ mg protein^−1^; H_2_ uptake: 433±7 µmol H_2_ min^−1^ mg protein^−1^).

Hydrogenase activity of the purified enzymes was also assayed for both H_2_ evolution and H_2_ uptake (Figure 5). Both activities are decreased by 2-fold at pH 8.0 in comparison to the WT, showing that the mutation does not cause a bias towards one of the two directions of catalysis.

In order to investigate if the effects observed on enzymatic activity are due to structural changes in the enzyme and to exclude that the functional features might be biased by aspecific structural distortions, the proteins were studied by circular dichroism (CD) spectroscopy and by absorbance spectroscopy.

**Figure 6:**
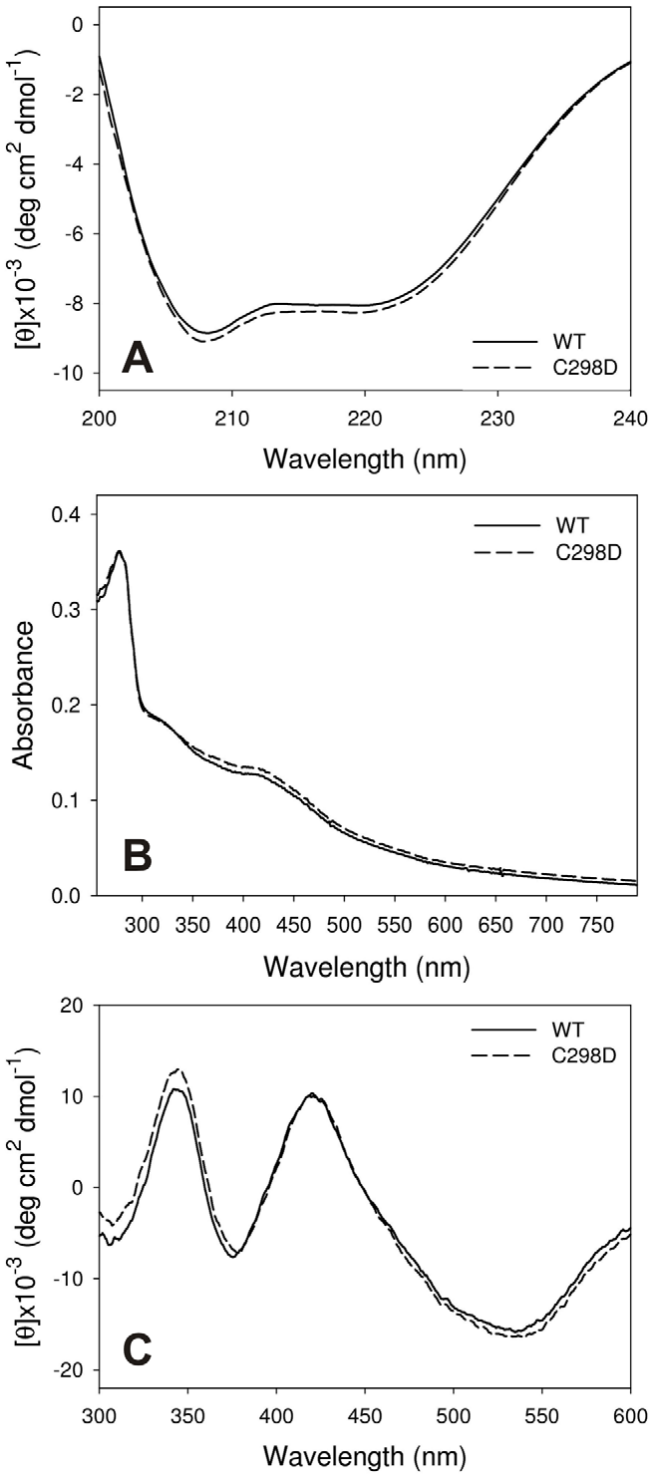
A) Far UV circular dichroism spectra of CaHydA WT (full line) and C298D (dashed line). Spectra were acquired under aerobic conditions in 50 mM Tris·HCl, 200 mM KCl, pH 8.0. C298D mutation does not cause significant differences in secondary structure. **B**) **UV-visible absorbance spectra of oxidised CaHydA WT (full line) and C298D (dashed line).** Spectra were acquired under aerobic conditions in 50 mM Tris·HCl, 200 mM KCl, pH 8.0. **C**) **Visible circular dichroism spectra of oxidised CaHydA WT (full line) and C298D (dashed line).** A different behaviour is observed between 250–300 nm, whereas the visible region does not show any difference, demonstrating identical insertion of iron sulphur clusters.

The far UV CD spectrum is not influenced by the mutation (Figure 6a), indicating that the replacement of the polar amino acid cysteine with negatively charged aspartic acid in the core of the protein does not cause significant changes in secondary structure.

Moreover, the UV visible absorbance spectrum (Figure 6b) and the visible CD spectrum (Figure 6c) are not significantly influenced by the mutation. In both WT and C298D, broad absorbance shoulders due to iron sulphur centres can be observed in the visible region at 322 nm and 418 nm. Cotton effects typical of clostridial hydrogenases [30] can be observed in the visible CD spectra in proximity of the absorbance shoulders. These results show that, although local structural rearrangements might be present, the iron content and the overall structure of the protein do not appear to be significantly altered by the mutation. Furthermore, the replacement of a highly conserved amino acid in the active site of CaHydA with a very different residue, still allows hydrogenase activity at high rates.

## Discussion

Only recently site directed mutagenesis and directed evolution have been applied to investigate the contribution of enzyme structure to important mechanistic properties of [FeFe]-hydrogenases including proton transfer [24], catalysis [19], gas diffusion and oxygen inactivation [23], [31]–[33].

Here the role of C298 in [FeFe]-hydrogenase activity is investigated because of its proximity to the active site and its putative role in proton transfer to the H-cluster. Using site saturation mutagenesis, a random approach that is a novel technique in [FeFe]-hydrogenase field, we demonstrated that aspartic acid is the only residue that can replace C298 with the highest efficiency.

Previous site directed mutagenesis works on CaHydA C298 or its analogous in other [FeFe]-hydrogenases (CrHydA1 C169, CpI C299) showed apparently contrasting conclusions. The mutant CrHydA1 C169S was previously demonstrated to have an H-cluster that was locked in the H_trans_ state and was catalytically inactive [19], in accordance with our results. CaHydA C298A and C298L showed a 6-fold loss of enzymatic activity (respectively 16% and 16.8% in hydrogen uptake) [23]. CpI C299A and C299S showed higher impairment: 20-fold and 6-fold respectively (6% and 16% in hydrogen uptake) [24]. In contrast with the latter observation, another report showed that CpI C299S was fully inactive [19]. These contrasting data can be explained because of different recombinant expression systems and different experimental conditions used to measure activity. It is reasonable that these variants were not identified in our screening because they were below or very close to the lower detection limit of the method of 14% WT activity. Nevertheless, the consensus is that any mutation of C298 that exchanges the thiol functional group leads to a strong decrease in catalytic activity.

Considering also that C298 is strictly conserved in all known [FeFe]-hydrogenases and in accordance with previous works, our results demonstrate that in this position cysteine has a major role in catalysis.

To clarify C298 role, it is interesting to consider in details the results of the library screening and the property of the various amino acids.

First of all, the only variant that sustains catalytic activity at high level is C298D. Given the statistical compliance and the sequencing validation of library completeness, our results confirm that the presence of any other amino acid in position 298, with the exception of cysteine and aspartic acid, severely impairs activity.

It is a remarkable fact that the C298S variant was identified among the negative clones, thus below our detection threshold; this confirms that C298S activity is highly impaired and this is probably because although serine is structurally related to cysteine, it is not functionally similar. The solution acidic constant (pK_a_) of the cysteine S-H group is ∼8.3, whereas for serine O-H this value is significantly higher and it is hardly ever dissociated at physiological pH. The C298A variant, which was reported in literature to have an activity impairment comparable to the C to S mutant, was also identified as a negative clone (Figure 3).

Moreover, C298N variant was identified among the negative clones, showing that an ionisable group is required to retain enzymatic function. Thus, aspartic acid (pK_a_ ∼3.9) can replace cysteine in this position, but the structurally similar but non-ionisable asparagine cannot.

The variants C298E and C298H resulted negative clones in our conditions. C298E carries a residue with a similar pK_a_ (∼4.3) but with longer side chain than aspartic acid. C298H, bears an ionisable side chain (pK_a_ ∼6.5) but has very different hindrance from both cysteine and aspartic acid. The presence of steric restrictions, together with the requirement of side chains pK_a_ values within a certain pH range, explains the fact that the only ionisable amino acid able to replace C298 is aspartic acid.

The fact that C298 can be replaced only by an ionisable amino acid is a strong evidence that C298 is a key point in proton transfer to the active site. In fact, it is known that proton transfer in several other proteins is mediated by water molecules and charged side chains of amino acids [34]; protons are transferred with a hopping mechanism by means of these ionisable chemical groups within proteins [35]. Moreover, an important role in proton transfer in the proximity of the active site was demonstrated for glutammic acid in [NiFe]-hydrogenases [36].

In this respect our work provides insights in the proton transfer pathway which are complementary to the recent study by Cornish *et al.* [24], where more external residues E282 and S319 were precisely assigned to be involved in proton transfer, while the internal cysteine was too buried to be targeted by azide rescue and Zn^2+^ inhibition assays.

Since no crystal structure is available for this protein, but a high sequence identity (70%) is observed with the CpI hydrogenase of known structure [10], [12], homology modelling can reliably offer some structural insights.

**Figure 7:**
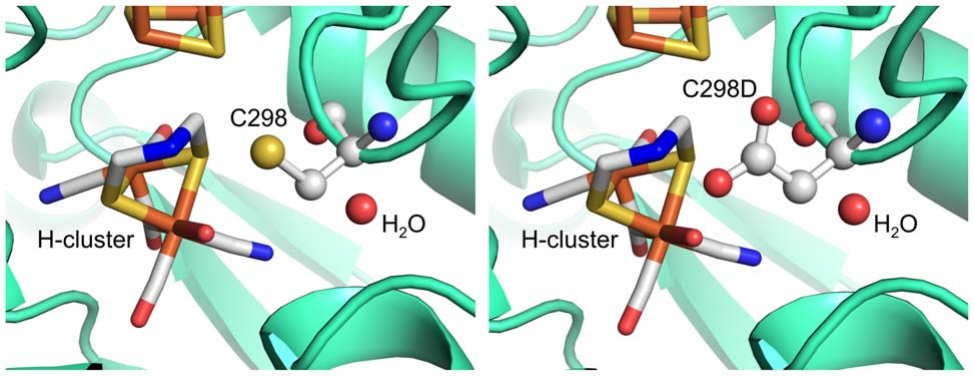
Model of CaHydA structure illustrating C298 role in catalysis. C298 mediates proton transfer from a molecule of structural water to the nitrogen atom of the active site (left). Aspartic acid can replace cysteine because of similar steric hindrance (right) and protonability, allowing both hydrogen evolution and uptake.

A comparison of models of the protein structure (Figure 7) shows that aspartic acid can fit structurally at position 298. Thus, the sulphur atom of cysteine would be replaced by the carboxylic group of aspartic acid, where it can participate in proton exchange between a nearby water molecule and a nitrogen atom at the bridgehead group of the H-cluster.

Experimentally, the C298D mutation does not have significant influence on the protein structure in comparison to WT: secondary structure was not altered, as demonstrated by far UV circular dichroism (CD) spectroscopy. In addition, the iron sulphur clusters were properly incorporated in the protein, as demonstrated both by visible CD spectroscopy and absorbance spectroscopy.

The lack of major structural differences ensures that C298D mutation does not affect protein folding or iron incorporation. Thus, observed functional differences are only due to the local effects of amino acid replacement.

Importantly, the fact that the oxygen inactivation rate is not influenced by the C298D mutation demonstrated that the replacement of cysteine 298 with aspartic acid does not cause a significant effect in oxygen diffusion to the active site. This is relevant because C298 delimitates a gas diffusion pathway from the protein surface to the active site [37], [23]. Moreover, C298D mutation does not directly influence the H-cluster reactivity with oxygen, a complex issue that is currently under study [38]–[40].

From the functional point of view, hydrogenase enzymatic activity of purified C298D was demonstrated both in the hydrogen evolution and uptake directions, confirming the results of the library screening.

Concerning H_2_ evolution activity, the small difference in *K*_M_ value for methyl viologen compared to the wild type enzyme was expected since C298D mutation most likely does not affect methyl viologen interaction with the protein, whereas the approximately 2-fold decrease in *k*_cat_ is probably the result of a slower rate in proton transfer involving the more acidic carboxylic group of aspartic acid at pH 8.0.

The shift of pH activity profile towards more acidic conditions is a major point in the demonstration of C298 role. Catalytic activity of the enzyme at different pH would be influenced only if the residue is part of the proton transfer pathway, because its intrinsic acidic properties would influence the proton exchange ability of the whole pathway. In facts, cysteine acidic constant (pK_a_∼8.3) is very different from that of aspartic acid (pK_a_∼3.9), even if it is known that actual pK_a_ of amino acids can be influenced by protein environment [33], [41] with shifts up to 4 pH unit from the pK_a_ measured in solution. In our case, it is likely that the pK_a_ of aspartic acid is shifted toward positive values while the cysteine pK_a_ is apparently shifted to more acidic values: this might be due not only to the effect of neighbour amino acids, but also of the water molecule involved in proton transfer [24]. Nonetheless the carboxyl group of aspartic acid is expected to be more easily deprotonated than the sulphydryl group of cysteine at low pH. The experimental evidence also highlighted that upon C to D mutation the only change of the Dixon-Webb Plot refers to the titration of a single protonable group shifted to a lower pK_a_ by about one unit, while the rest of the profile is unchanged.

Thus, differences in the pH activity profile between WT and C298D mutant demonstrate that C298 is part of the proton transfer pathway to the active site, as when it is replaced by a more acidic residue the activity is strongly shifted towards more acidic pH. Moreover, as kinetic curves were measured at pH 8.0, this difference partially explains the difference observed in *k*_cat_ values.

Since C298 is strictly conserved in all [FeFe]-hydrogenases, it is reasonable that our observations in CaHydA are valid also for other enzymes of this class, probing a common mechanism in hydrogenase catalysis. As a preliminary result, *in vivo* activity assays, performed in our laboratory on *Chlamydomonas reinhardtii* CrHydA1 recombinantly expressed in *E. coli,* confirmed that in this algal [FeFe]-hydrogenase the conserved cysteine corresponding to C298 can be mutated to an aspartic acid residue still retaining a high enzyme activity.

In conclusion, we demonstrated that cysteine 298 (C298) in CaHydA [FeFe]-hydrogenase can be efficiently replaced only by aspartic acid. The identification of such an unexpected structural replacement was obtained by the use of random mutagenesis approach. Importantly, C298D mutation does not cause any severe structural change. Hydrogen evolution activity is conserved with a 2-fold decrease in *k*_cat_ only and a severe shift in pH activity profile by 1 pH unit towards more acidic conditions is observed in comparison with WT. The exclusive replacement of cysteine with an ionisable residue and the shift in pH activity clearly demonstrate the central role of C298 in the proton transfer pathway to the active site during [FeFe]-hydrogenase catalysis.

## Materials and Methods

### Site saturation library preparation

The library was prepared using the QuikChange strategy with in-house adapted protocols [26]. Parental plasmid pCaE2 harbouring the wild type gene was amplified by PCR using the two degenerate primers CCTATGTTTACATCTNNKTGTCCTGCATGGGTA (forward) and TACCCATGCAGGACAMNNAGATGTAAACATAGG (reverse) with KOD Hot Start DNA Polymerase (Merck); PCR product was digested with DpnI restriction enzyme (Fermentas) to eliminate parental DNA and immediately transformed into chemically competent *E. coli* DH5α cells. Resulting clones were collected together and the mixture of mutated plasmids was sequenced to demonstrate NNK randomization (where N =  any nucleotide, K = G or T) of the desired positions.

### Library screening by *in vivo* activity assay

*E. coli* BL21(DE3) chemically competent cells were co-transformed with the hydrogenase plasmid library and with the maturation plasmid pCaFG. Single colonies were transferred on M63-agar plates supplemented with antibiotics, 1.5 mM IPTG and 5 mM methyl viologen (MV); plates were sealed in a glass jar and anaerobiosis was set up by flushing pure argon. After over night incubation at 30°C to allow IPTG-induced expression of the hydrogenase, enzymatic activity (hydrogen uptake) was detected by flushing pure hydrogen for 30 minutes. Colonies expressing an active hydrogenase quickly developed a blue colour due to specific reduction of MV. Selected variants were identified by DNA sequencing. Negative controls were performed using colonies transformed with plasmids lacking the hydrogenase gene. Sensitivity threshold controls were performed using colonies transformed with a plasmid containing a mutated and characterized hydrogenase gene encoding for a variant with an impared activity (14% of WT uptake activity and 18% of WT hydrogen evolution activity) in the purified form.

### CaHydA WT and C298D recombinant expression and purification

*Clostridium acetobutylicum* HydA hydrogenase (CaHydA) and C298D mutant were recombinantly produced in the active form in *E. coli* with the adaptation of protocols previously described [42]. Briefly, *E. coli* BL21(DE3) chemically competent cells were co-transformed with two plasmids: 1) pCaE2 harbouring the hydA gene and the maturation gene hydE and 2) pCaFG harbouring the maturation genes hydF and hydG from *C. acetobutylicum.* After aerobic growth, expression was induced with 1.5 mM IPTG and the cells were incubated over night under anaerobic conditions at 30°C into sealed bottles. To prevent hydrogenase inactivation by atmospheric oxygen, all following manipulations, including cell harvesting, purification, storage and assay were carried out under strict anaerobic conditions, using a glove box (Plas Labs) with a hydrogen/nitrogen atmosphere. All solutions were supplemented with 2–20 mM sodium dithionite, vacuumed and finally equilibrated with the glove box atmosphere prior to use. CaHydA WT and C298D were affinity purified with Strep-Tactin Superflow high capacity cartridges (IBA) following manufacturer's instructions. Coomassie-stained SDS-PAGE was routinely used to assess purity of the enzymes. Protein concentration was assayed with Bradford assay using bovine serum albumin as standard. The typical yield was 2.5 mg pure protein/L culture. The same procedure described above was applied for expression and purification of the sensitivity control variant employed in the screening for estimating the lowest detectable activity.

### Hydrogenase activity assays

All assays were performed at 37°C in 50 mM Tris·HCl pH 8.0 at least in triplicate. Hydrogen evolution activity was assayed by gas chromatography with 10 mM reduced methyl viologen as artificial electron donor. Reactions were set up into sealed headspace vials (20 mL), filled with 2 mL reaction mixture. Anaerobiosis was obtained by flushing pure argon. Methyl viologen was reduced by the anaerobic addition of at least 2-fold excess of sodium dithionite. The reaction was started by anaerobic addition of the purified enzyme (final concentration was in the nM range). The gas was sampled with a SampleLock Gastight syringe (Hamilton) and analyzed by gas chromatography. The gas chromatographer (Agilent Technologies 7890A) was equipped with purged packed inlet, Molesieve 5A column (30 m, ID 0.53 mm, film 25 µm) and thermal conductivity detector; argon was used as carrier gas. Efficient and quantitative separation was achieved in less than 3 min at 60°C. Hydrogen uptake activity was assayed spectrophotometrically with H_2_-saturated buffer and 10 mM oxidised methyl viologen as artificial electron acceptor. Reduction of methyl viologen was followed at 604 nm using a molar extinction coefficient of 13.6 mM^−1^ cm^−1^.

### Hydrogen evolution kinetics and pH activity profile

Hydrogen evolution kinetic curves were obtained as described above using reduced methyl viologen as artificial electron donor in the range 1–40 mM. Resulting data were plotted and fitted to Michaelis-Menten hyperbolic curve. Activity profiles at different pH were measured in 50 mM MES·NaOH (pH range 5.0–6.5) or 50 mM ACES·NaOH (pH range 6.5–7.5) or 50 mM Tris·HCl (pH range 7.5–9.0) using 10 mM dithionite-reduced methyl viologen as artificial electron donor; ionic strength was fixed to 50 mM with NaCl. Non-linear fittings to a 3-parameter sigmoid curve for WT and C298D *k*_cat_ values dependence on pH in the range pH 5–8 were performed with Sigma-Plot.

### Oxygen inactivation assay

The assay was performed in accordance to previously described method [43]. Briefly, aliquots of enzyme (approximately 7 µM) in 100 mM Tris·HCl, 150 mM NaCl pH 8.0 were placed in gastight vials. At zero time the rubber stopper was removed and the samples were exposed to air at room temperature. At intervals (after 0, 5, 7.5, 10 and 12.5 min), samples were removed and residual activity was measured by hydrogen uptake assays.

### Spectroscopy

All spectroscopy experiments were carried out in 50 mM Tris·HCl, 200 mM KCl, pH 8.0 under aerobic conditions in the absence of dithionite. Absorbance spectra were acquired with an Agilent 8453E UV-Vis spectrometer. Circular dichroism spectra were acquired with a Jasco J-815 CD spectrometer using a 1 mm path cell (far UV) or a 1 cm path cell (visible). Spectra are the average of 3 accumulations. Molar ellipticity was calculated using the residue average molecular weight 110.9 Da for the far UV region and the protein molecular weight 65519 Da for the visible region.

### Structural modelling

A model of CaHydA structure was built using the Swiss-Model server with CpI structure as template (pdb 3C8Y) [12]. Non-protein atoms were manually added and the model was explored with the software PyMol.

## References

[1] Adams MWW (1990) The structure and mechanism of iron-hydrogenases. Biochim Biophys Acta 1020: 115–145. 2173950 10.1016/0005-2728(90)90044-5

[2] Vignais PM, Billoud P (2007) Occurrence, classification, and biological function of hydrogenases: an overview. Chem Rev 107: 4206–4272. 17927159 10.1021/cr050196r

[3] Hallenbeck PC (2009) Fermentative hydrogen production: principles, progress, and prognosis. Int J Hydrogen Energy 34: 7379–7389.

[4] McKinlay JB, Harwood CS (2010) Photobiological production of hydrogen gas as a biofuel. Curr Opin Biotechnol 21: 244–251. 20303737 10.1016/j.copbio.2010.02.012

[5] Cracknell JA, Vincent KA, Armstrong FA (2008) Enzymes as working or inspirational electrocatalysts for fuel cells and electrolysis. Chem Rev 108: 2439–2461. 18620369 10.1021/cr0680639

[6] Hambourger M, Gervaldo M, Svedruzic D, King PW, Gust D, et al (2008) [FeFe]-hydrogenase-catalyzed H_2_ production in a photoelectrochemical biofuel cell. J Am Chem Soc 130: 2015–2022. 18205358 10.1021/ja077691k

[7] Armstrong FA, Belsey NA, Cracknell JA, Goldet G, Parkin A, et al (2009) Dynamic electrochemical investigations of hydrogen oxidation and production by enzymes and implications for future technology. Chem Soc Rev 38: 36–51. 19088963 10.1039/b801144n

[8] Fontecilla-Camps JC, Volbeda A, Cavazza C, Nicolet Y (2007) Structure/function relationships of [NiFe]- and [FeFe]-hydrogenases. Chem Rev 107: 4273–4303. 17850165 10.1021/cr050195z

[9] Meyer J (2007) [FeFe] hydrogenases and their evolution: a genomic perspective. Cell Mol Life Sci 64: 1063–1084. 17353991 10.1007/s00018-007-6477-4 PMC11136429

[10] Peters JW, Lanzilotta WN, Lemon BJ, Seefeldt LC (1998) X-ray crystal structure of the Fe-only hydrogenase (CpI) from *Clostridium pasteurianum* to 1.8 Angstrom resolution. Science 282: 1853–1858. 9836629 10.1126/science.282.5395.1853

[11] Nicolet Y, Piras C, Legrand P, Hatchikian CE, Fontecilla-Camps JC (1999) *Desulfovibrio desulfuricans* iron hydrogenase: the structure shows unusual coordination to an active site Fe binuclear center. Structure 7: 13–23. 10368269 10.1016/s0969-2126(99)80005-7

[12] Pandey AS, Harris TV, Giles LJ, Peters JW, Szilagyi RK (2008) Dithiomethylether as a ligand in the hydrogenase H-Cluster. J Am Chem Soc 130: 4533–4540. 18324814 10.1021/ja711187e

[13] Silakov A, Wenk B, Reijerse E, Lubitz W (2009) ^14^N HYSCORE investigation of the H-cluster of [FeFe] hydrogenase evidence for a nitrogen in the dithiol bridge. Phys Chem Chem Phys 11: 6592–6599. 19639134 10.1039/b905841a

[14] Nicolet Y, Fontecilla-Camps JC, Fontecave M (2010) Maturation of [FeFe]-hydrogenases: Structures and mechanisms. Int J Hydrogen Energy 35: 10750–10760.

[15] Mulder DW, Shepard EM, Meuser JE, Joshi N, King PW, et al (2011) Stepwise [FeFe]-hydrogenase H-cluster assembly revealed in the structure of HydA^ΔEFG^ . Structure 19: 1038–1052. 21827941 10.1016/j.str.2011.06.008

[16] De Lacey AL, Fernández VM (2007) Activation and inactivation of hydrogenase function and the catalytic cycle: spectroelectrochemical studies. Chem Rev 107: 4304–4330. 17715982 10.1021/cr0501947

[17] Lubitz W, Reijerse E, van Gastel M (2007) [NiFe] and [FeFe] Hydrogenases Studied by Advanced Magnetic Resonance Techniques. Chem Rev 107: 4331–4365. 17845059 10.1021/cr050186q

[18] Fontecilla-Camps JC, Amara P, Cavazza C, Nicolet Y, Volbeda A (2009) Structure-function relationships of anaerobic gas-processing metalloenzymes. Nature 460: 814–822. 19675641 10.1038/nature08299

[19] Knörzer P, Silakov A, Foster CE, Armstrong FA, Lubitz W, et al (2012) Importance of the Protein Framework for Catalytic Activity of [FeFe]-Hydrogenases. J Biol Chem 286: 38341–38347. 10.1074/jbc.M111.305797 PMC3256906 22110126

[20] Nicolet Y, Lemon BJ, Fontecilla-Camps JC, Peters JW (2000) A novel FeS cluster in Fe-only hydrogenases. Trends Biochem Sci 25: 138–143. 10694885 10.1016/s0968-0004(99)01536-4

[21] Greco C, Bruschi M, De Gioia L, Ryde U (2007) A QM/MM Investigation of the Activation and Catalytic Mechanism of Fe-Only Hydrogenases. Inorg Chem 46: 5911–5921. 17602468 10.1021/ic062320a

[22] Hong G, Cornish AJ, Hegg EL, Pachter R (2011) On understanding proton transfer to the biocatalytic [Fe-Fe]_H_ sub-cluster in [Fe-Fe] H2ases: QM/MM MD simulations. Biochim Biophys Acta 1807: 510–517. 21296047 10.1016/j.bbabio.2011.01.011

[23] Lautier T, Ezanno P, Baffert C, Fourmond V, Cournac L, et al (2011) The quest for a functional substrate access tunnel in FeFe hydrogenase. Faraday Discuss 148: 385–407. 21322495 10.1039/c004099c

[24] Cornish AJ, Gärtner K, Yang H, Peters JW, Hegg EL (2011) Mechanism of Proton Transfer in [FeFe]-Hydrogenase from *Clostridium pasteurianum* . J Biol Chem 286: 38341–38347. 21900241 10.1074/jbc.M111.254664 PMC3207428

[25] Chica RA, Doucet N, Pelletier JN (2005) Semi-rational approaches to engineering enzyme activity: combining the benefits of directed evolution and rational design. Curr Opin Biotechnol 16: 378–384. 15994074 10.1016/j.copbio.2005.06.004

[26] Reetz MT, Carballeira JD (2007) Iterative saturation mutagenesis (ISM) for rapid directed evolution of functional enzymes. Nat Protoc 2: 891–903. 17446890 10.1038/nprot.2007.72

[27] Patrick WM, Firth AE, Blackburn JM (2003) User-friendly algorithms for estimating completeness and diversity in randomized protein-encoding libraries. Protein Eng 16: 451–457. 12874379 10.1093/protein/gzg057

[28] Dixon M, Webb EC (1979) Enzymes, 3^rd^ ed. New York: Academic Press.

[29] Cleland WW (1982) The use of pH studies to determine chemical mechanisms of enzyme-catalyzed reactions. Methods Enzymol 87: 390–405. 7176923 10.1016/s0076-6879(82)87024-9

[30] Multani JS, Mortenson LE (1972) Circular dichroism spectra of hydrogenase from *Clostridium pasteurianum* W5. Biochim Biophys Acta 256: 66–70. 5009821 10.1016/0005-2728(72)90163-6

[31] Stapleton JA, Swartz JR (2010) A Cell-Free Microtiter Plate Screen for Improved [FeFe] Hydrogenases. PLoS ONE 5: e10554. 20479937 10.1371/journal.pone.0010554 PMC2866662

[32] Stapleton JA, Swartz JR (2010) Development of an In Vitro Compartmentalization Screen for High-Throughput Directed Evolution of [FeFe] Hydrogenases. PLoS ONE 5: e15275. 21151915 10.1371/journal.pone.0015275 PMC2997796

[33] Bingham AS, Smith PR, Swartz JR (2012) Evolution of an [FeFe] hydrogenase with decreased oxygen sensitivity. Int J Hydrogen Energy 37: 2965–2976.

[34] Gunner MR, Mao J, Song Y, Kim J (2006) Factors influencing the energetics of electron and proton transfers in proteins. What can be learned from calculations? Biochim Biophys Acta 1757: 942–968. 16905113 10.1016/j.bbabio.2006.06.005 PMC2760439

[35] Cukierman S (2006) Et tu, Grotthuss! and other unfinished stories. Biochim Biophys Acta 1757: 876–885. 16414007 10.1016/j.bbabio.2005.12.001

[36] Dementin S, Burlat B, De Lacey AL, Pardo A, Adryanczyk-Perrier G, et al (2004) A Glutamate Is the Essential Proton Transfer Gate during the Catalytic Cycle of the [NiFe] Hydrogenase. J Biol Chem 279: 10508–10513. 14688251 10.1074/jbc.M312716200

[37] Cohen J, Kim K, King P, Seibert M, Schulten K (2005) Finding gas diffusion pathways in proteins: application to O_2_ and H_2_ transport in CpI [FeFe]-hydrogenase and the role of packing defects. Structure 13: 1321–1329. 16154089 10.1016/j.str.2005.05.013

[38] Goldet G, Brandmayr C, Stripp ST, Happe T, Cavazza C, et al (2009) Electrochemical Kinetic Investigations of the Reactions of [FeFe]-Hydrogenases with Carbon Monoxide and Oxygen: Comparing the Importance of Gas Tunnels and Active-Site Electronic/Redox Effects. J Am Chem Soc 131: 14979–14989. 19824734 10.1021/ja905388j

[39] Stripp ST, Goldet G, Brandmayr C, Sanganas O, Vincent KA, et al (2009) How oxygen attacks [FeFe] hydrogenases from photosynthetic organisms. PNAS 106: 17331–17336. 19805068 10.1073/pnas.0905343106 PMC2765078

[40] Lambertz C, Leidel N, Havelius KGV, Noth J, Chernev P, et al (2011) O_2_ Reactions at the Six-iron Active Site (H-cluster) in [FeFe]-Hydrogenase. J Biol Chem 286: 40614–40623. 21930709 10.1074/jbc.M111.283648 PMC3220472

[41] Pace CN, Grimsley GR, Scholtz JM (2009) Protein Ionizable Groups: pK Values and Their Contribution to Protein Stability and Solubility. J Biol Chem 284: 13285–13289. 19164280 10.1074/jbc.R800080200 PMC2679426

[42] King PW, Posewitz MC, Ghirardi ML, Seibert M (2006) Functional studies of [FeFe] hydrogenase maturation in an *Escherichia coli* biosynthetic system. J Bacteriol 188: 2163–2172. 16513746 10.1128/JB.188.6.2163-2172.2006 PMC1428129

[43] Adams MWW, Mortenson LE (1984) The physical and catalytic properties of hydrogenase II of *Clostridium pasteurianum* . J Biol Chem 259: 7045–7055. 6327705

